# The impact of traditional and religious practices on the spread of Ebola in West Africa: time for a strategic shift

**DOI:** 10.11694/pamj.supp.2015.22.1.6190

**Published:** 2015-10-10

**Authors:** Angellar Manguvo, Benford Mafuvadze

**Affiliations:** 1 Department of Medical Education Support Services, School of Medicine, University of Missouri-Kansas City, USA; 2 Department of Pathology, University of Kansas Medical Center, USA

**Keywords:** Ebola, traditional and religious practices, traditional leaders, spiritual healers

## Commentary

The current Ebola viral disease outbreak in West Africa, affecting communities in Guinea, Liberia, and Sierra Leone with contained cases in Nigeria, Mali, and Senegal, is the largest known outbreak since the discovery of the virus in 1976. Several factors including the emergence of the disease in highly populated urban areas, poor health facilities, and a general lack of awareness of the disease among affected communities perpetuated the spread of the disease. In addition, researchers [1–4] concur that the widespread embracing of certain traditional and religious practices among West African communities had tremendous negative effects on the spreading of the disease. In concurrence, the World Health Organization (WHO) [5] contents that nearly 60% of all Ebola cases reported in Guinea can be linked to traditional burial practices.

Scientifically-based methods of combatting the spread of highly infectious diseases like Ebola are normally preferred, with very little considerations given to the impact of traditional and religious practices on preventive measures [6]. However, recent trends in West Africa have demonstrated that the use of scientific methods alone without a holistic consideration of other contextual factors is not sufficient to control the disease. For example, an analysis of media reports and recent studies [7] in West Africa reveals notable resistance against prescribed scientific ways of combating the transmission of Ebola in some affected communities. While there is no single explanation for this kind of resistance, as this is a very complex social phenomenon, the influence of religious and cultural beliefs cannot be denied. It is, thus, important that we recognize the impact that traditional and religious practices can have on health promotion interventions. It is also important that we acknowledge ethnic and cultural diversity among communities. For example, in Liberia, there are at least 16 major ethnic and cultural groups; each described by a specific language and associated traditions and customs [8]. Understanding these ethno-social dynamics should help policy-makers with formulation of disease prevention approaches that are culturally tolerated by affected communities.

Given the challenges faced with ongoing efforts to contain the spread of Ebola that arise as a result of incompatibilities between some religious and cultural practices and prescribed scientific methods, it is high time we explore ways in which traditional and religious structures can be effectively implored in combatting the spread of Ebola. Dealing with cultural and religious issues negatively affecting Ebola transmission prevention programs requires flexibility and adaptability from governments, health officials, and the people in affected communities. As Alexander et al. [9] argue, a consideration of traditional and religious practices is critical to our understanding of transmission dynamics and subsequent control of highly infectious diseases. In this paper, we identified notable religious and traditional practices in West Africa that potentially contribute to the spread of Ebola and exacerbate the current outbreak. Our proposed strategies call for deliberate targeting of leaders of traditional and religious institutions in community-directed programs for preventive measures. As custodians of the day-to-day cultural values, traditional and religious leaders command more respect and authority in their communities than unfamiliar trained health personnel, who can be easily be viewed as having suspicious agendas.

### The Impact of religion and tradition on transmission of Ebola

***Perceived Causation of Diseases and Death:*** adherence to prescribed preventive measures by people in affected communities is central to a desirable outcome in the fight against Ebola. A general lack of understanding of disease etiology can, however, negatively influence level of adherence to preventive measures [10]. In the West African context, diseases and death are generally perceived as a culmination of natural and metaphysical causes [11]. Metaphysical causes entail the spiritual realm such as witchcraft and punishment from God or ancestral spirits for breaking taboos and various forms of transgressions [11]. Undoubtedly, this line of thinking can potentially make it difficult for ordinary people in affected communities to fully understand the cause of Ebola-related deaths as a viral infection.

Furthermore, West Africa is home to some of the world's fastest-growing Islamic and Christian communities. Both the Bible and the Koran abound with verses and suras that dwell on causality of infectious diseases. For example, commonly cited verses include Deuteronomy 28 verses 20-22 where it is stated that, as a consequence of various transgressions, God strikes people with diseases, fever, and inflammation. Others have falsely cited the occurrence of Ebola as God-inflicted punishment for indulgence in activities like adultery and homosexuality [12]. The way people conceptualize the etiology of a disease generally dictates their response to it. Given that in some affected communities in West Africa, Ebola is linked to the metaphysical realm, it is not surprising that diviners and spiritual healers are often consulted for treatment. In view of the potential influence of etiological beliefs on people's response to prevention and treatment, an understanding of beliefs of the people in affected communities on causes of Ebola is pivotal in mitigating the negative impact of such beliefs on the transmission of the disease.

***Traditional and Spiritual Healing:*** while modern health care based on Western medicine is now considered the norm in Africa, several communities in West Africa still rely heavily on traditional medical practices. According to WHO [13], about 70-80% of the population in some West African countries depend on traditional medicine. As noted by Wonacott [14], traditional healers are often more highly regarded than those who promote unfamiliar forms of health care. Whereas traditional medicine can have a positive role in health care, some ethno-medical beliefs and practices, as discussed earlier, can also have important negative impact on health outcomes and, most importantly, pathogen transmission pathways. For example, studies on the current Ebola outbreak in West Africa revealed that several traditional and spiritual healers falsely claimed to have the capability to cure Ebola [3]. One reported case was the employment of warm salt baths and salt drinks provided by traditional and spiritual healers as a preventive measure from contracting the highly infectious disease [3]. A previous study by Hewlett and Hewlett [15] reported similar claims by traditional healers in Uganda who were making incisions into people's bodies and rubbing herbal medicine in. Given that human-to-human transfer of Ebola occurs from direct contact with body fluids of the infected, it is not surprising that several people including traditional healers themselves reportedly got infected through these practices. By falsely claiming capability to treat and rid patients of Ebola, traditional healers are often called to attend to severely sick patients. Without proper protective resources to cater for the sick Ebola patients, it is not surprising that most of the traditional healers often end up contracting the disease themselves and passing it to their other patients. It is with this recognition that Guinea's head of Ebola Response Team had to dissuade a delegation of traditional healers against using their formula for fighting the lethal virus [14].

The media has also reported of several faith healers who prayed for Ebola victims and not only ended up contracting the diseases themselves but passing the disease to other people [16]. For example, it was reported that several high-ranking members of a church contracted and died from Ebola after they participated in a healing prayer session in which they laid hands on the body of a sick person [17]. Most churches have since abolished this practice of laying hands on the sick as a measure to prevent the spread of the disease. Both Christian and Islamic leaders can, thus, play a significant role in educating their institutional members on the importance of strict adherence to preventive measures for combatting the spread of Ebola.

Overall, reports of attempts to cure Ebola through traditional and spiritual means highlight the need for increased cooperation between traditional healers, spiritual healers, and trained health personnel. We propose that awareness programs specifically targeting traditional and spiritual healers in affected communities and all high risk areas be launched once an outbreak of Ebola has been reported. Without fully understanding the etiology, pathogenesis, transmission vehicles, as well as signs and symptoms of the disease, it becomes difficult for traditional and spiritual healers to distinguish potential Ebola patients from patients with other conditions that they can cure. Furthermore, without proper understanding of the disease, traditional and spiritual healers are often sources of false information and myths to the general public particularly in remote rural areas where there is limited access to information through electronic and print media. We further propose that traditional and spiritual healers form regional and national associations through which regular collaborative workshops with health professionals can be organized with the ultimate goal of creating a mutual understanding. Such programs will not only help reduce the spread of Ebola but will also be important in dealing with several other health issues. Recent reports that some traditional healers in Sierra Leone openly acknowledged their ignorance of the Ebola virus and called on other healers to suspend healing activities until they are given adequate training [1] show a willingness to embrace modern scientific knowledge. Trained traditional healers can, thus, potentially be used as personnel to train other traditional and spiritual healers and dispel myths on the etiology, pathogenesis, transmission, as well as prevention of Ebola.

***Funeral and Burial Practices:*** most communities in West Africa believe in life after death [18]. Consequently, funeral and burial practices are given a lot of significance as they are perceived as crucial steps in transitioning from the world of the living to the spiritual world [18]. It is widely believed that the transition should be facilitated by the surviving relatives through funeral and burial rituals. In the event that the deceased fails to attain the more elevated rank of ancestral spirit, it is believed that their spirit may return and punish the living relatives. One of the most commonly performed funeral rituals, which significantly contributes to the spread of Ebola, is the washing and cleaning of the dead body. Another reported burial practice is that of relatives of the deceased washing their hands in a common bowl after which they touch the face of the deceased in what is perceived as a ‘love touch’ that cements unity between the living and ancestral spirits [19]. In the case of the death of prominent people like traditional healers, it is common for people to lay over the corpse with the hope that some of the spiritual gifts will be transferred to them [19]. Given that the major means of human-to-human transfer of the Ebola virus is through direct contact with infected body fluids as consistently reported in the current Ebola outbreak, the afore-mentioned funeral and burial practices inadvertently result in spreading of the disease [4]. A previous study in Uganda showed that a number of people who contracted Ebola during the 2003 outbreak potentially acquired the virus in the process of performing similar burial rituals [15]. In spite of the obvious negative impact of the burial practices on the spread of Ebola, most West African communities, as mentioned earlier, place significant value on the rituals and resist adoption of alternative methods that minimize the spread of the virus such as cremation. Against this background, the questions that arise when outbreaks of a disease like Ebola occur are: how do we make sure that people desist from practices of getting into contact with the deceased's body?; who should monitor and ensure that people do not continue with such practices?; how do we ensure proper burial of the deceased in line with cultural norms but minimizing the chances of contracting the disease?; should we completely abandon cultural burial practices and enforce scientifically prescribed methods that presumably reduce the spread of the disease?

In an effort to reduce the spread of Ebola through burial practices, WHO, in conjunction with affected governments set guidelines defining how bodies of Ebola victims are to be handled and subsequently buried [20]. While the guidelines were formatted in line with current scientific knowledge of the disease, it is obvious that not much attention was given to the cultural implications of some of the prescribed measures to the affected communities. Some of the measures, such as cremation, are in direct conflict with widely held views of life after death as previously discussed. It may sounds easy for people in other cultures to think that people in West Africa should realize that the highly infectious nature of the disease and its high mortality rates require extraordinary measures to contain; However, it is also important to realize that some of these funeral practices span across generations and have been practiced in these communities for centuries. Moreover, in an effort to quickly dispose dead bodies and reduce spread of the disease, some ‘Dead Body Management Teams’, often carried out burials prior to notifying relatives of the deceased [9]. Given the sacred obligations given to the deceased and the value placed on funeral traditions, as mentioned earlier, this created suspicion and discontentment amongst affected communities. Resultantly, in an attempt to avoid having their relatives cremated or buried without their consent, some of the people did not report their deceased relatives and secretly continued with traditional burial practices and, in the process, thwarted efforts to combat the spreading of Ebola [16]. Moreover, there were several reports in Liberia of corpse collectors allegedly accepting bribes to provide death certificates falsely validating that the victim did not die of Ebola, allowing the funeral practices to continue [16]. In recognition of the unpopularity of cremation, the Liberian government recently called off the cremation decree and ordered the establishment of special burial plots for the deceased victims of Ebola [21]. Although WHO guidelines emphasized the need for understanding and respecting cultural practices and religious beliefs, according to experts [7], the major challenge is for health officers to convince involved communities to desist from inherent cultural practices.

It is, therefore, our proposition that traditional leaders should be consulted in order to modify cultural rituals to levels that are effective in prevention of disease but still culturally acceptable. Awareness campaigns specifically targeting traditional leaders such as chiefs, village headmen, and spiritual leaders should be launched once an outbreak has been confirmed and in all high risk areas. As custodians and enforcers of traditional customs in society, it is easier for people to respect orders that contradict their own values when instructions are given by well-respected leaders as compared to unfamiliar health officers. As evidenced in some of the affected communities, misinformed traditional leaders can hinder preventive efforts. On the other hand, well-informed traditional leaders can potentially play crucial roles in reducing the spread of Ebola. In addition, well-informed traditional leaders can help with identification of people in their communities who can be trained and provided with protective equipment for burial of suspected Ebola victims in specially-designated burial areas in line with their cultural norms. In a similar manner, Christian and Islamic leaders can draw on guidelines described in the Bible and the Koran respectively, to encourage their congregants to adhere to scriptural recommendations of handling of dead bodies that minimize the contraction of infectious diseases. For example, the book of Numbers (Chapter 19, Verses 11-12) describes how people were to handle themselves after touching a dead body. Similarly, in Leviticus (Chapter 21, Verses 10-12), priests were instructed not to come in contact with any dead person probably as a way of preventing them from spreading infections to other members of the community given their prominent role in society. Through highlighting such verses, both Christian and Islamic leaders can play a significant role in educating their fellow congregants on the importance of reducing contact with deceased bodies.

## Conclusion

The current Ebola outbreak in West Africa clearly demonstrates that scientifically proven methods of combating the transmission of infectious diseases, if not culturally and religiously acceptable to a community, are likely to be resisted and rendered less effective. There is, thus, a need to investigate and align preventative measures with cultural norms and values of affected communities. Where there are incompatibilities between religious and cultural practices and prescribed scientific preventive measures, there is a need to widely consult and collaborate with traditional and religious leaders. We propose that, for effectiveness of community-based preventive programs, traditional leaders should be engaged and assigned important roles in monitoring and implementation of preventive measures against the spread of Ebola.

One of the major challenges faced with the current Ebola outbreak is lack of mutual trust and understanding between health officials and affected communities. The mistrust was particularly worsened when burials were sometimes implemented without relatives’ consent. The rapid burial of deceased without notification of relatives resulted in a belief that medical professionals were keeping the corpses for nefarious purposes. In a similar outbreak in Uganda in 2003, such mistrust was exacerbated when rumors circulated that some Westerners were buying human body parts [15]. Fostering mutual trust between health officials and affected communities is the ultimate key to success of scientific measures aimed at combatting the spread of the disease. Without this mutual trust, adherence to preventive measures is likely to be compromised and, as witnessed in some communities, health official were even attacked and barred from executing their duties. The inclusion of traditional and spiritual leaders and trained people drawn from affected communities in leadership positions is likely to improve understanding and adherence to preventive measures. As Alexander et al. [9] argue, most people in West African communities often look to traditional leaders for advice in times of catastrophic strikes; as such, traditional and spiritual leaders can have a significant influence on effective implementation of preventive measures against the spread of Ebola.

**Figure 1:**
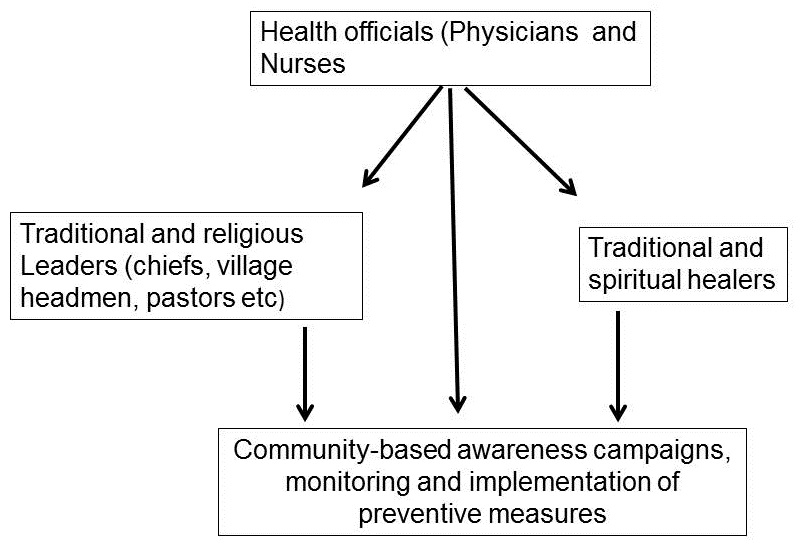
Proposed approach to dealing with traditional and religious issues impacting preventive measures in Ebola affected areas. Once an outbreak is confirmed or suspected, apart from making all health personnel (physicians, nurses, assistants et cetera), awareness campaigns specifically targeting traditional and religious leaders (chiefs, village headmen, church pastors, mosque imams), as well as traditional and spiritual healers should be launched. These groups of people should then collaborate in educating, implementation and monitoring of prescribed preventive measures. Traditional leaders should identify people under their jurisdiction that will be trained and assist in carrying out community-based preventive programs such as burial of deceased bodies

There is a likelihood that widespread Ebola vaccination campaigns will be launched in the near future. Previous polio vaccination campaigns in Nigeria were boycotted by some communities amidst rumors that the vaccine contained infertility drugs, caused polio-myelitis, and spread HIV and AIDS, in addition to varied religious reasons [22]. It is, thus, likely that the success of Ebola vaccination campaigns in West Africa will, to a large extent, depend on the direct involvement of traditional and religious leaders. We further identified the need for awareness campaigns specifically targeting traditional and spiritual healers. Given the religious and cultural beliefs of some people in West Africa, it is difficult to effectively control spread of Ebola without the support of traditional and spiritual healers. It is encouraging to note that a recent study by Umeora [3] shows that some traditional and spiritual healers and leaders have shown some willingness to collaborate with health officials in mobilizing communities for effective prevention of the spread of Ebola. Figure 1, summarizes our proposition that awareness campaigns specifically targeting traditional leaders, traditional and spiritual healers, and other influential community leaders should be launched in all high risky areas for effective implementation of Ebola awareness and preventive measures. As highlighted in this paper, it is crucial to consult leaders of traditional and religious institutions and collaborate with them at all stages of Ebola prevention campaigns especially those that are incompatible with cultural and religious practices.
